# Serum and cerebrospinal fluid immune mediators in children with autistic disorder: a longitudinal study

**DOI:** 10.1186/s13229-016-0115-7

**Published:** 2017-01-05

**Authors:** Carlos A. Pardo, Cristan A. Farmer, Audrey Thurm, Fatma M. Shebl, Jorjetta Ilieva, Simran Kalra, Susan Swedo

**Affiliations:** 1 Johns Hopkins University School of Medicine, 627 Pathology Bld., 6000 North Wolfe Street, Baltimore, MD 21287 USA; 2 Pediatrics and Developmental Neuroscience, National Institute of Mental Health, Bethesda, MD USA; 3 Yale School of Public Health, Yale University, New Haven, CT USA

**Keywords:** Autism, Immune, CSF, Chemokine, Cytokine, Growth factor

## Abstract

**Background:**

The causes of autism likely involve genetic and environmental factors that influence neurobiological changes and the neurological and behavioral features of the disorder. Immune factors and inflammation are hypothesized pathogenic influences, but have not been examined longitudinally.

**Methods:**

In a cohort of 104 participants with autism, we performed an assessment of immune mediators such as cytokines, chemokines, or growth factors in serum and cerebrospinal fluid (*n* = 67) to determine potential influences of such mediators in autism.

**Results:**

As compared with 54 typically developing controls, we found no evidence of differences in the blood profile of immune mediators supportive of active systemic inflammation mechanisms in participants with autism. Some modulators of immune function (e.g., EGF and soluble CD40 ligand) were increased in the autism group; however, no evidence of group differences in traditional markers of active inflammation (e.g., IL-6, TNFα, IL-1β) were observed in the serum. Further, within-subject stability (measured by estimated intraclass correlations) of most analytes was low, indicating that a single measurement is not a reliable prospective indicator of concentration for most analytes. Additionally, in participants with autism, there was little correspondence between the blood and CSF profiles of cytokines, chemokines, and growth factors, suggesting that peripheral markers may not optimally reflect the immune status of the central nervous system. Although the relatively high fraction of intrathecal production of selected chemokines involved in monocyte/microglia function may suggest a possible relationship with the homeostatic role of microglia, control data are needed for further interpretation of its relevance in autism.

**Conclusions:**

These longitudinal observations fail to provide support for the hypothesized role of disturbances in the expression of circulating cytokines and chemokines as an indicator of systemic inflammation in autism. ClinicalTrials.gov, NCT00298246.

**Electronic supplementary material:**

The online version of this article (doi:10.1186/s13229-016-0115-7) contains supplementary material, which is available to authorized users.

## Background

Autism spectrum disorder (ASD) is a lifelong neurodevelopmental disorder found in up to 1% of the US population [1], characterized by social communication deficits and restricted and repetitive behaviors [2]. Risk for ASD is highly associated with genetic factors [3], but current evidence suggests that neurobiological abnormalities in ASD are associated with changes in cytoarchitectural and neuronal organization that may be determined by the complex interplay of genetic, environmental, and immunological mechanisms [4–7]. Although ASD is not a classical immune-mediated disorder, there is increasing interest in examining the role of the immune system and inflammation in the development and persistence of the complex neurological and behavioral abnormalities associated with ASD [8–10].

Both innate and adaptive branches of the immune system are involved in critical mechanisms of brain development, neuronal and cortical organization, developmental and adaptive synaptic plasticity, and critical stages of brain function that determine neurological and behavioral activity into adulthood [11]. From fetal development to adulthood, the immune system and central nervous system (CNS) establish interactions which can influence both systemic immune responses (peripheral immune system) and local CNS immune function (neuroimmunity). Several lines of research have revealed abnormalities in the interactions of the immune system and CNS in ASD which involve disturbances in both adaptive and innate immunity [4, 6, 12, 13].

Evidence from the effects of maternal viral infections during pregnancy [14], an excess of autoimmune disorders in mothers of subjects with ASD or their families [15, 16], and the effects of environmental factors on the formation of the immune system [17] support the view that various types of disturbances of immune function play roles in the pathogenesis of ASD and the perpetuation of associated behavioral and neurological abnormalities. Studies of immune function in ASD frequently have focused on functional and quantitative studies of T cells and B cells, antibody production, and the presence of autoantibodies against neuronal epitopes. Studies of cellular immunity found abnormal function and number of T cells, as well as a lower percentage of CD4 T cells and a skewed CD4:CD8 T cell ratio [9]. However, these studies involved small samples, and more recent studies have demonstrated altered function in selected immune pathways and cell systems such as T regulatory cells and NK cells in subjects with ASD [18–20].

An emerging theme centers on the role of cytokines and chemokines, immune mediators which play important roles in pro-inflammatory or anti-inflammatory responses in the periphery and CNS. Cytokines and chemokines play important immune, homeostatic, and regulatory roles in the microenvironment of specific tissues or systems as well as circulating blood and immune cell populations [21–24]. Measures of circulating immune mediators or the rate of production by specific immune cell populations under specific conditions have been used for assessment of immune reactivity status including pro-inflammatory stages during disease. Although measurement of specific cytokines or chemokines lack specificity to define disease, their profiles and spectrum of expression may help to understand pathogenic mechanisms of disease in which the immune system may be involved [25–30]. Interestingly, cytokines and chemokines also may facilitate processes of immune-CNS interactions which modulate not only inflammatory responses within the CNS but also neuroimmune mechanisms associated with neuronal homeostasis, synaptic plasticity, and neuroglial function [31]. However, the literature is inconsistent in that no single marker is found to be abnormal consistently across studies. The majority of existing studies in ASD characterized cytokine and chemokine profiles in the plasma or serum [32], and each study finds a different profile of differences between children with ASD and children with developmental delay or typical development, most commonly elevations in ASD [33–36].

Fewer studies have examined the CNS directly, through the cerebrospinal fluid (CSF) or brain tissues; Vargas and colleagues [37] found increased levels of pro-inflammatory and modulatory cytokines, differentially expressed across the cortical regions in post-mortem samples from individuals with ASD. The goal of this study was to provide data testing the hypothesis that children with ASD differ in immune profile, especially in pro-inflammatory markers, from those without ASD. Given the importance of determining the role of immune-CNS interactions in ASD, the current study was designed to longitudinally assess immune mediators in children with ASD compared to children with typical development by focusing on the expression profile of cytokine and chemokine networks in the peripheral immune compartment (serum), to determine the pattern of expression of such immune mediators in the neuroimmune system compartment represented by CSF and to assess the correspondence between peripheral and neuroimmune system compartments.

## Methods

### Participants and assessment

Participants were drawn from a longitudinal study of autism (NCT00298246) implemented at the National Institute of Mental Health/NIH, Bethesda, Maryland. A legal guardian provided written consent for participation in this NIH Combined Neurosciences Institutional Review Board-approved study (06-M-0102). In the autism group (AUT), autistic disorder was diagnosed using DSM-IV-TR [38] criteria by a team of doctoral-level experienced clinicians, using the Autism Diagnostic Interview-Revised [39], Autism Diagnostic Observation Schedule [40], and clinical judgment. Typically developing controls (TYP) were recruited for lack of concern in any domain of development and were screened using the Social Communication Questionnaire [41] and cognitive testing. The groups were not matched on cognitive function, and correlation between immune profile and clinical characteristics was outside the scope of the current report. All children in the current study were between the ages of 2 and 7.99 at baseline and were followed at intervals of approximately one year, for up to 3 years. Per study design, serum samples were collected from subjects up to four times at intervals to approximately 1 year, and CSF samples were collected once (at baseline) or twice (at final visit, if possible).

Medical history related to immune status (i.e., allergies, immunodeficiency, autoimmune disorder) was collected for both groups of children via structured interview with medical personnel. For members of the AUT group who underwent lumbar puncture, serum was collected contemporaneously with CSF, under sedation, following a 12-hour fasting period. All CSF and blood samples (including those collected in the absence of lumbar puncture) were collected during the morning period (9 am–12 noon). Ethical constraints prevented lumbar punctures in the TYP group, so none of the TYP serum samples were collected under sedation. CSF was immediately centrifuged after the LP procedure, and acellular aliquots were stored at −70 °C within 20–30 min. Serum samples were collected following standard procedures and aliquoted and stored within 20–30 min after separation. Up to four serum samples and up to two CSF samples were obtained from participants, at intervals ranging from 9–24 months, and stored until simultaneous laboratory analysis.

### Assay technique

We used multiplexed bead assay techniques for establishing the profiles of 39 immune mediator proteins that included cytokines, chemokines, and growth factors in serum and CSF. The selected panel was the most comprehensive available. Assay reagents and plates were obtained from well-validated commercial sources (Millipore®) [42]. The procedures followed recommendations and well-established protocols for evaluation of serum [42, 43] and the CSF [44, 45]. Only the first freeze-thaw aliquots were used for assay measurements. To achieve uniformity in the longitudinal assessment, assays for samples collected at different timepoints from the same individual were run simultaneously. Masked samples were measured in duplicates and blank values subtracted from all readings. Measurements and data analysis of all assays were performed with the Luminex-200® system in combination with Luminex manager software (Bioplex manager 5.0, Bio-Rad, Hercules, CA). We used standard operating procedures to guarantee the consistency, reproducibility, and reliability of the assays. Samples that exhibited unexpected or unacceptable variance (i.e., evidence of bead clumping, coefficients of variation greater than 20%, or unusual distributions of values) were re-tested.

### Statistical analysis

#### Serum

Data analysis was performed in SAS/STAT Version 9.3 [46] (PROC MIXED or PROC GLIMMIX). The design of this analysis was roughly accelerated longitudinal case-control, which allows the study of change over a long interval of age by gathering data over shorter, variable periods of time, from participants with baselines staggered across the age range of interest. Modern statistical methods, particularly those that utilize maximum likelihood estimation, are well-suited to analyzing these data. Mixed models with restricted maximum likelihood estimation are commonly used for longitudinal data [47], using chronological age as the time metric (rather than time point), and refer to the “mixed” use of random and fixed effects. In this case, the models included a random intercept, which accounts for the correlation resulting from the up to four samples within the same subject during study participation. Each of the models also included several fixed effects, which were sex, diagnosis, and chronological age. The best-fitting model from those evaluating quadratic change with age, linear change with age, or no change with age was selected. We then considered the main effect of diagnosis (i.e., does the mean level of the analyte differ between groups?), and in the case of quadratic or linear change models, the interaction of diagnosis and age (i.e., does the difference between diagnoses depend on age?). Raw *p* values were used at the model-fitting stage of analysis to find the best candidate model, but false discovery rate (FDR; [48]) adjusted *p* values were used to determine final statistical significance (<.05) and are reported.

Within-subject estimates of stability in serum concentrations were obtained using the ratio of variance explained by the subject cluster to the total variance, controlling for age, and replacing out-of-range values with the limit of detection in order to provide the most conservative estimate. These estimated intraclass correlation coefficients (ICCs) range from 0 to 1 and are interpreted as standard correlation coefficients (i.e., values from .60 to .80 are considered moderate, ≥.80 are considered strong).

Concentrations were transformed with a natural logarithm, and values outside the range of detection were set to missing. Variables with more than 30% out-of-range values were analyzed as categorical (detected versus not), in which case the generalized linear mixed model with binomial distribution and a logit link function was implemented in the strategy described above.

Finally, although the use of maximum likelihood estimation to address missing data is commonplace, we conducted sensitivity analyses in which the out-of-range values were imputed with the limit of detection (e.g., all out-of-range EGF values were replaced with 2.7 pg/mL) and all variables were treated as continuous.

#### CSF

CSF was available only for a subset of the AUT, and two samples were obtained for a minority of participants. Therefore, change over time in CSF was not modeled with mixed models of change in the analyte across the age range, but instead, general stability estimates (correlation) for sample 1 and sample 2 (controlling for age and time-to-follow-up) were generated. For analytes with an overall rate of out-of-range values less than 30%, partial Spearman’s correlations were used, and logistic regression was used for variables with high out-of-range rates. The relationship between circulating and CSF levels of the immune modulators was assessed by calculating the fraction of intrathecal production (also called percentage transfer), which is the ratio of CSF concentration of a specific protein (immune mediator) to serum concentration (percentage transfer = [Immune mediator_CSF_/immune mediator_Serum_] × 100). A fraction of intrathecal production value greater than 100% indicates higher levels of production within the CNS relative to the periphery compartment; conversely, values less than 100% suggest the analyte production is mostly in the periphery with minimal production within the CNS [49]. Finally, the Spearman correlation between serum and CSF values were calculated, as it is possible that analytes with rates of production that differ between compartments may still be correlated.

## Results

Participants were 104 children diagnosed with DSM-IV-TR Autistic Disorder (AUT) and 54 typically developing controls (TYP), aged 2–7.99 years at initial evaluation (Table 1). Serum samples were obtained in all AUT and TYP subjects while CSF was obtained in 67 AUT subjects. No participant had a history of immunodeficiency or autoimmune disorder. Food, environmental, and seasonal allergies were present in a minority of participants, but were more common in AUT (*n* = 36, 35%) than in TYP (*n* = 10, 19%) (*χ* ^2^ = 4.46, *p* = .03). Basic peripheral blood and immune features (i.e., white blood cell count, IgG, IgM, IgA) did not differ between groups (Additional file 1: Table S1). Basic composition of the CSF appeared normal; white blood cell count was normal, and the CSF to serum albumin concentration quotient, which is an index of blood-CSF barrier integrity, was within age-adjusted expectations [50].

**Table 1:** Participant demographic characteristics (for sample 1)

	Full sample		Subsample with CSF
	AUT	TYP	AUT
*N*	104	54	67
Male, *n* (%)	86 (83)	41 (76)	55 (82)
Race, *n* (%)			
Black	20 (19)	4 (7)	12 (18)
Asian	4 (4)	0	3 (5)
White	74 (71)	44 (81)	47 (70)
Multiple races	5 (5)	5 (9)	4 (6)
Unknown	1 (1)	1 (2)	1 (2)
Ethnicity, *n* (%)			
Hispanic	7 (7)	4 (7)	4 (6)
Non-Hispanic	97 (93)	49 (90)	63 (94)
Unknown	0	1 (2)	0
Age, *M ± SD*	4.41 ± 1.27	3.64 ± 1.11	3.60 ± 0.95
Full scale DQ, *M ± SD*	50.49 ± 18.04	109.91 ± 12.90	53.42 ± 16.32
Body mass index	16.68 ± 2.21	16.53 ± 1.42	16.52 ± 1.51
Number of samples, *n* (%)			
1	104 (100%)	54 (100%)	67 (100%)
2	82 (78)	32 (59)	31 (46)
3	37 (36)	25 (46)	--
4	11 (11)	6 (11)	--
Parent reported immunologic history^a^, *n* (%)			
None	68 (65)	44 (81)	43 (64)
Allergies (food, environmental, seasonal)	36 (35)	10 (19)	24 (36)
Immunodeficiency or autoimmune disorder	0	0	0
Serum basic features, *M ± SD*			
WBC count	8.15 ± 2.31	7.16 ± 1.73	8.42 ± 2.37
IgG, mg/dL	838.07 ± 251.71	760.94 ± 188.37	774.52 ± 252.22
IgM, mg/dL	83.95 ± 35.52	84.89 ± 33.78	80.73 ± 37.48
IgA, mg/dL	88.05 ± 51.82	86.81 ± 46.77	78.48 ± 46.26
CSF basic features, *M ± SD*			
WBC count	–	–	0.79 ± 1.03
Albumin quotient	–	–	2.62 ± 1.60

Data missing for two participants in each group.

Note: Sample size differed slightly for basic laboratory features. Serum WBC count: AUT, *n* = 101; TYP, *n* = 52. Serum IgG/IgM/IgA: AUT, *n* = 95; TYP, *n* = 52. CSF WBC count, *n* = 63. CSF albumin quotient: *n* = 61. Body mass index sample size was AUT (serum), *n* = 85; TYP, *n* = 47; AUT (CSF), *n* = 64

### Serum immune mediators

Descriptive data for each of the serum immune mediators are presented in Additional file 1: Table S2A and S2B.

Thirty of 39 analytes were analyzed as continuous variables; the remaining had 30% or more values outside the range of detection and were analyzed as categorical variables (detected versus not detected). Results of the mixed models are presented in Table 2; variables analyzed as categorical are denoted with a superscript a. This table is interpreted as follows: the effect of age is shown in the Age or Age^2^ column, depending on which was the best-fitting model according to the uncorrected *p* values. For linear or quadratic trends with uncorrected *p* values <.05, between-group differences in slope were tested with a contrast statement (none were significant, *p* values are not reported). Evidence for linear change over age (the Age column of Table 2) was found in IL12p40, TNFα, and CCL22 (MDC); the general trend was for a decrease in the analyte across ages 2 to 8 years, and these patterns did not differ between groups (i.e., the Contrast statements were non-significant). There was no evidence for change over age in the remaining analytes.

**Table 2:** Serum results of mixed models

	Limit of detection (pg/mL)	% outside limit of detection	Within *df*	Test statistics from mixed model		
				Group (1 *df*)	Age (2 *df*)	Age^2^ (2 *df*)
				*F(p)*	*F(p)*	*F(p)*
Cytokines						
IL-1α	0.35	15	149	0.01 (.94)		
IL-1RA^a^	0.09	49	193	0.27 (.85)		
IL-1β^a^	0.03	49	193	0.23 (.85)		
IL-2	0.03	29	117	0.46 (.81)		
sIL-2RA^a^	0.30	33	189	0.43 (.81)	7.72 (.01)	3.65 (.13)
IL-3^a^	0.01	46	193	4.80 (.13)		
IL-4	0.04	28	116	0.59 (.77)		
IL-5	0.01	10	163	0.2 (.86)		
IL-6^a^	0.06	34	193	0.23 (.85)		
IL-7	0.03	12	156	0.89 (.70)		
IL-9	0.01	6	171	0.03 (.93)		
IL-10	0.01	27	119	0.1 (.88)	3.97 (.11)	4.5 (.07)
IL-12p40	0.57	16	146	1.12 (.60)	5.81 (.04)	
IL-12p70	0.05	15	147	1.27 (.60)		
IL-13^a^	0.01	37	193	0.62 (.77)		
IL-15^a^	0.17	52	193	0.40 (.81)		
IL-17	0.01	5	177	0.10 (.88)		
IFNα2	0.12	5	174	0.79 (.73)		
IFNγ	0.04	2	186	0.02 (.93)		
TNFα	na	0	190	0.02 (.93)	13.10 (<.001)	
TNFβ	0.02	27	123	0.04 (.93)		
TGFα	0.02	12	153	1.12 (.60)		
Growth factors						
EGF	2.70	7	170	8.64 (.04)		
G-CSF	1.65	1	186	0.10 (.88)	3.99 (.11)	
GM-CSF	1.85	2	186	0.36 (.81)		
VEGF	3.36	3	185	2.26 (.43)		
FGF-2	4.08	8	166	0.02 (.93)		
FLT-3L^a^	0.04	57	193	1.19 (.60)		
sCD40L^a^	1 671 700	53	193	15.0 (<.001)		
Chemokines						
CCL2 (MCP-1)	na	0	192	2.04 (.44)		
CCL3 (MIP-1α)	0.03	8	169	1.59 (.53)		
CCL4 (MIP-1β)	2.9	1	191	0.15 (.88)		
CCL7 (MCP-3)	0.73	21	132	0.59 (.77)		
CCL11 (EOTAXIN)	17.06	1	188	0.01 (.94)		
CCL22 (MDC)	na	0	190	4.73 (.13)	7.92 (.01)	
CXCL1 (GRO)	na	0	193	1.65 (.53)		
CXCL8 (IL-8)	na	0	189	0.12 (.88)	1.63 (.53)	5.18 (.07)
CXCL10 (IP-10)	na	0	192	2.59 (.42)		
CX3CL1 (FRACTALKINE)	2.1	26	119	0.02 (.93)		

Note: *df* = degrees of freedom; na = not applicable. Out-of-range values were below the limit of detection for all variables except sCD40L, for which out-of-range refers to values above the limit of detection. Out-of-range values were set to missing, except where the % outside the limit of detection was ≥30%. Those variables were analyzed as categorical (out-of-range versus detectable), modeled with a binary distribution and logit link. A series of nested models were tested; sequential models for quadratic, linear, and no effect of age were tested. Where a linear or quadratic term was significant, a contrast statement was used to determine the difference in linear slope or quadratic shape between groups; in no case was this contrast statistically significant. Raw *p* values were used to determine the best-fitting model; FDR-adjustment was performed after this model was selected and are presented in the table; many terms did not remain significant after correction. All models included sex as a covariate and a random effect of intercept to control for observations correlated within subject

^a^Variables that were analyzed as categorical (out-of-range versus detectable) and modeled with a binary distribution and logit link. The remaining variables were analyzed as Ln-transformed continuous variables with a Gaussian distribution

The Group column reflects the test of whether a mean difference existed between groups. A significant mean difference between groups (the Group column in Table 2) was observed on EGF (AUT > TYP, Cohen’s *d* = 0.44, 95% CI = 0.11–0.77), and a significant difference in odds of out-of-range values was observed for sCD40L (OR = 3.40, 95% CI = 1.82–6.33; AUT was more likely to be above the range of detection than TYP). Neither of these effects was diminished when history of allergies, body mass index, or platelet count was added to the model. No significant differences in other analytes were observed.

Results of the sensitivity analyses, wherein out-of-range values were imputed at the limit of detection and all variables were assessed as continuous, were consistent with the primary analysis (data available upon request).

**Fig. 1:**
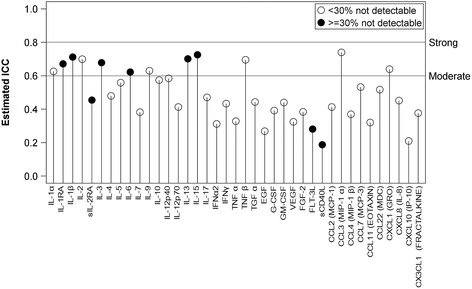
Estimated intraclass correlations (ICC) for each variable. The estimated ICC was obtained using the ratio of variance explained by the subject cluster to the total variance, controlling for age (out-of-range values were replaced with the limit of detection). Lower values indicate less variance explained by subject cluster, or less within-subject correlation or stability. By convention, values above .60 are considered moderate and above .80 are considered strong

Generally, the estimated ICC values for serum were low, indicating a lack of within-subject stability (Fig. 1). ICC values ranged from .19 (sCD40L) to .73 (CCL3/MIP-1α), but most fell well below .60 (the median value was .45). Both analytes found to differ, on average, between groups had low ICC (sCD40L, ICC = .19; EGF, ICC = .27).

### CSF immune mediators

Descriptive data for each of the CSF immune mediators are presented in Additional file 1: Table S3. Unlike serum, repeated CSF samples were available for a minority of participants (*n* = 31). Rather than modeling change over age as was done in serum, we calculated the correlation between CSF sample 1 and sample 2, controlling for age (*M ± SD* = 3.51 ± 0.95 years) and time-to-follow-up (*M ± SD* = 2.44 ± 0.68 years, range 1.17 to 3.53 years). Sample 1 values were significantly related to sample 2 values for most analytes (Table 3). The rate of out-of-range values for sample 2 given an out-of-range value for sample 1 ranged from 50–100%.

**Table 3:** Estimated stability of CSF immune mediators in children with autism, controlling for age and time-to-follow-up

	Partial Spearman correlations for variables with <30% outside the LOD			% <LOD for one or both samples	Rate of sample 2 <LOD where sample 1 <LOD
	*n*	Partial correlation, 95% CI	*p* value		
Cytokines					
IL-1A	26	.33 (−.09–.64)	.11	16	1/5 (20%)
IL-IRA				87	21/25 (84%)
IL-1B				87	23/27 (85%)
IL-2				84	20/23 (87%)
sIL-2RA				65	15/17 (88%)
IL-3				52	15/15 (100%)
IL-4				97	29/29 (100%)
IL-5	28	.85 (.68–.93)	<.0001	10	1/3 (33%)
IL-6				94	28/29 (97%)
IL-7				71	19/19 (100%)
IL-9	31	.82 (.63–.91)	<.0001	0	na
IL-10				35	8/11 (73%)
IL-12p40				61	13/19 (68%)
IL-12p70				94	24/25 (96%)
IL-13				100	30/31 (97%)
IL-15	31	.80 (.61–.90)	<.0001	0	na
IL-17				94	27/28 (96%)
IFNα2	30	.74 (.50–.87)	<.0001	3	0/1 (0%)
IFNγ				68	16/18 (89%)
TNFα	31	.62 (.32–.80)	.004	0	na
TNFβ				65	12/16 (75%)
TGFα	31	.84 (.67–.92)	<.0001	0	na
Growth factors					
EGF				100	31/31 (100%)
G-CSF	31	.53 (.19–.75)	.003	0	na
GM-CSF	31	.72 (.48–.86)	<.0001	0	na
VEGF				97	26/29 (90%)
FGF-2				74	17/21 (81%)
FLT-3L	31	.82 (.64–.91)	<.0001	0	na
sCD40L	27	.63 (.30–.82)	.0008	13	1/4 (25%)
Chemokines					
CCL2 (MCP-1)	31	.64 (.35–.81)	.0002	0	na
CCL3 (MIP-1α)				58	9/13 (69%)
CCL4 (MIP-1β)				48	12/12 (100%)
CCL7 (MCP-3)				71	12/19 (63%)
CCL11 (EOTAXIN)				97	26/30 (87%)
CCL22 (MDC)				32	5/10 (50%)
CXCL1 (GRO)	29	.53 (.18–.75)	.004	6	0/2 (0%)
CXCL8 (IL-8)	31	.51 (.17–.74)	.004	0	na
CXCL10 (IP-10)	31	.50 (.16–.73)	.01	0	na
CX3CL1 (FRACTALKINE)	31	.42 (.06–.68)	.02	0	na

Note: <LOD = below the lower limit of detection; na = not applicable. No restrictions were placed on time-to-follow-up for inclusion in these analyses. Mean age for sample 1 was 3.51 ± 0.95 years; mean time-to-follow-up was 2.44 ± 0.68 years. Spearman’s correlation was calculated where at least 70% of the sample had detectable values at both visits. Listwise deletion was used in correlation analyses; thus, individuals with out-of-range values were excluded. Age at sample 1 and time-to-follow-up were partialled out in correlation analyses. Variables with high rates of values outside the range of detection were analyzed as categorical; because of the small sample size and complete or quasi-complete separation, it was not possible to calculate reliable odds ratio estimates for these variables

**Fig. 2:**
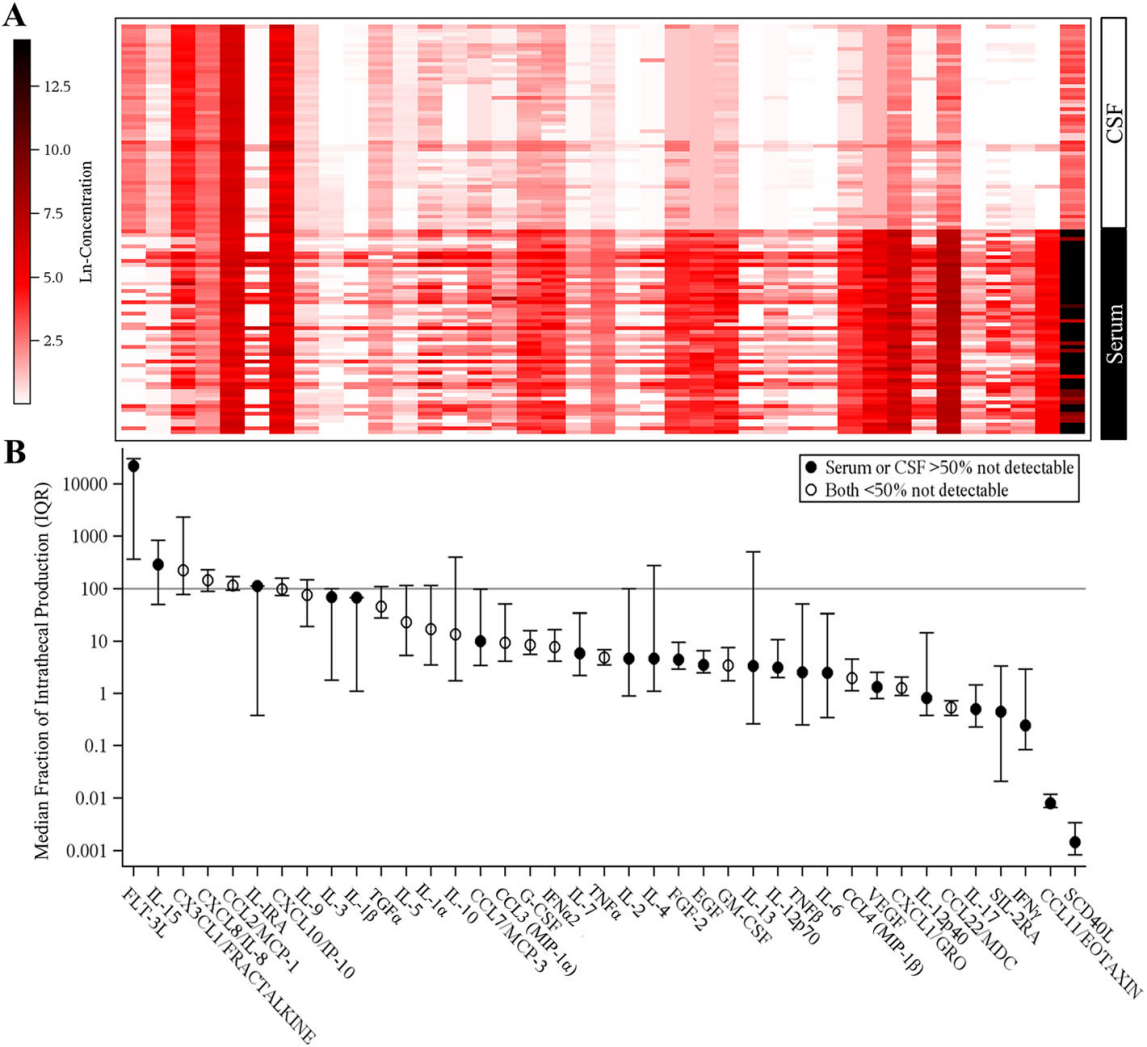
Relative concentrations and percent transfer (CSF:Serum) in AUT group with contemporaneous CSF and serum samples (*n* = 54). **a** The relative ln-transformed concentration of analytes (labeled in the Y axis of (**b**)) in CSF versus serum. **b** The median percent transfer (with interquartile range, IQR) for each analyte. For ease of presentation, Y axis units in (**b**) are log_10_. Values outside the range of detection were imputed with the limit of detection. Percent transfer values of 100% (*gray horizontal line*) reflect equal CNS and serum production

Fraction of intrathecal production rates were observed at both extremes (sCD40L, median 0.0008%; FLT-3L, median 22,000%) (Fig. 2). The vast majority of analytes had fraction of intrathecal production rates less than 100%, indicating relatively low CNS production compared to serum. With a few exceptions, correlations between serum and CSF values were low or non-significant, further indicating little relationship between the compartments (Additional file 1: Table S4).

## Discussion

The present study describes a comprehensive longitudinal study of serum and CSF profiles of immune mediators and modulators in children with ASD. Three key points emerge from these data. First, we used rare, longitudinal CSF sampling to demonstrate striking differences in the expression of selected cytokines, immune-related growth factors, and chemokines in the CSF compartment compared to the circulating bloodstream compartment. These findings are consistent with data from other populations [51], which show that peripheral immune-related proteins do not mirror the neuroimmune and CNS microenvironment. Second, we used these unique longitudinal data to demonstrate the limitations of cross-sectional data. Although our analyses revealed few developmental trends in circulating peripheral immune-related proteins during childhood, we noted a striking lack of within-subject stability in most of the analytes. The low ICC estimates obtained in this study indicate that a single measurement is not a reliable longitudinal indicator of a child’s level of most of serum immune mediators and modulators we studied. Third, we found no evidence for major differences in the expression of circulating cytokines and chemokines between children with autism and typically developing controls. The results of our study do not support the hypothesis that an active systemic inflammatory process plays a role in the persistence of autistic disorder and suggest that previously observed increases in brain tissue cytokines and chemokines [37, 52] may reflect homeostatic non-inflammatory roles in response to CNS dysfunction [10].

An overview of basic immune parameters such as leukocyte count and profile of circulating immunoglobulins revealed no differences between AUT and TYP. In the peripheral immune system, as reflected by the circulating bloodstream, only proteins with both immune modulation and growth factor functions (EGF and sCD40L) were significantly elevated longitudinally in AUT compared to TYP. These differences occurred in the absence of within-subject stability, suggesting that while there was significant within-subject variability, the general range of values observed in AUT differed from that of TYP across timepoints.

Interestingly, proteins such as TNF-α, IL-6, and IFNγ and chemokines such as CCL2 and CXCL10, frequently associated with pro-inflammatory responses, did not differ between AUT and TYP. The group differences observed in two of the 39 analytes tested in this study, elevated circulating EGF and sCD40L, could be construed as suggesting that dysregulation of growth and modulatory pathways, rather than systemic inflammatory responses, may occur in autism. In the periphery, both EGF and sCD40L are carried by platelets and involved in mechanisms of tissue repair, vascular function, and immune modulation [53]. EGF is a growth factor that plays critical roles in the growth, proliferation, and differentiation of numerous cell types [54, 55], and is involved in several pathways of neuronal function and trophism [55, 56]. The results of the current study are consistent with other comparisons of serum EGF between children with and without autism. Using enzyme-linked immunosorbent assay (ELISA), one study documented significantly elevated serum EGF in 27 Turkish children with autism aged 2–11 years relative to age-matched typical controls [57]. A second report, using multiplex bead assay, found elevated serum EGF in 77 children and adolescents with autism (aged 5–15) compared to 19 healthy, slightly older, controls [35]. However, some studies have documented decreased EGF in the plasma of subjects with autism compared to controls [58–60]. These conflicting results may be related to the type of sample used for the assays, as serum assays reflect all growth factors released by platelets during the blood sampling processing for obtaining serum [53]. It is worth noting that in the current study, group differences remained when platelet count was added to the model, though platelet count was not available for all TYP participants.

Similar to EGF, the CD40 system plays a regulatory role in the immune and vascular systems. sCD40L modulates function of B cells including stimulation of activation-associated surface antigen, immunoglobulin isotype switching, immunoglobulin secretion, and lymphocyte memory generation. The interaction of sCD40L with its receptor, CD40, also plays important function in monocyte activation and dendritic cell maturation [61]. No data exist regarding sCD40L in ASD, though one relevant study documented decreasing levels of sCD40L in healthy individuals across neonatal, childhood, and adult age groups [62]. We found no changes in sCD40L levels across childhood, but high rates of out-of-range values may have obscured any developmental trend. The second study, comparing children with Prader-Willi syndrome to healthy siblings, found no significant difference between the groups in plasma sCD40L, though the trend was for higher values in the syndromic children [63]. There are several studies documenting elevated levels of sCD40L in adult psychiatric and health conditions, where a negative relationship between sCD40L and cognitive function has been demonstrated [64, 65].

Thus, the significance of the elevated blood circulating levels of EGF and sCD40L in our participants with autism remains unknown, especially given the high degree of within-subject variability in concentration. Future investigations could determine whether they influence the spectrum of CNS, neurobehavioral, and cognitive dysfunction in subjects with ASD, but the variable nature of these analytes dictates that this should be done only with extreme caution, in longitudinal samples, and with appropriate controls to demonstrate the specificity to ASD (rather than for general developmental disability, for example). One hypothesis is that genetically determined growth or immune-modulatory dysregulation, rather than active systemic inflammatory responses, is responsible for elevated EGF and sCD40L. Previous SNP analyses of growth factor genes revealed a haplotypic association of EGF with ASD [66], suggesting that genetic factors may cause elevated EGF levels. An alternative hypothesis is that growth pathway dysregulation during early development pre-established a context of elevated EGF. However, our findings of low stability over time of EGF (and most other analytes) necessitate caution in considering an immune endophenotype.

A major finding of the present study is the lack of correspondence in the profiles of circulating immune-related modulators in the blood and CSF of participants with ASD. This incongruence highlights essential differences in the CNS/neuroimmune and peripheral immune system environments [67, 68]. Importantly, EGF and sCD40L, the two serum analytes on which AUT and TYP differed, had extremely low fraction of intrathecal production rates, indicating that peripheral activity had little bearing on central presence of these analytes. The relatively high levels and increased fraction of intrathecal production of immune mediators such as FLT3L, IL-15, CX3CL1, CXCL8, and CCL2 likely reflect the specific production of these mediators by neuroglia or neuronal cell populations in the CNS environment. The CSF profile of these immune mediators likely reflects a homeostatic role, as most of them have selective function on microglia and the neuroglia-neuronal interactions required for maintaining CNS homeostasis. For example, FLT3L promotes cell differentiation, proliferation, and survival and influence in the function of microglia cells in the CNS. Similarly, CX3CL1 and CCL2, chemokines that facilitate migration of monocytes to areas of injury, are critical for monocytes physiology and migration and play critical roles in the homeostatic function of microglia [69–71]. Interestingly, IL-15, a widespread expressed cytokine which is involved in multiple signaling pathways in the CNS of neurogenesis and neuroplasticity, is also a potent microglia modulator [72–74]. These observations suggest that the CNS environment in our population of subjects with ASD was enhanced in favor of microglia function rather than other adaptive neuroinflammatory responses, perhaps in response to a persistent stage of developmental synaptic plasticity or neurodevelopmental processes in the brain of these subjects [75].

Although this study focused on the assessment of immune mediators in the blood and CSF compartments, these results do not totally preclude the possibility that systemic immune factors or inflammation may influence brain development and alter neurobiological trajectories and subsequent long-term neurobehavioral changes. Such effects may only be observable when the immune markers are derived from specific microenvironment niches, or when they result from specific immune cell populations in response to exogenous challenges (e.g., isolated PBMC [76]). In those cases, the immune response is highly influenced by genetic determinants of the host, but its relevance to pathogenesis of autism remains uncertain. Regardless, the within-subject stability should be considered in future explorations of non-CSF compartment individual markers or profiles of these markers, as the high degree of variability of most analytes necessitates caution in interpreting single observations as an indicator of an enduring endophenotype. In addition, although CSF analyses permit a more direct window into the physiological status of the CNS than do peripheral measures, even they are not capable of providing information about neuroimmune responses occurring in selected areas of the brain, as has been demonstrated in previous studies of brain tissues [37].

One limitation of this study was the lack of comparison data for the CSF, as ethical constraints on lumbar punctures for healthy children precluded a comparison group in the present study. Little is known about potential differences between children and adults in CSF concentrations of immune mediators; still, comparison to adult samples may be helpful for context. AUT CSF concentrations of the six immune mediators with the highest percent transfer were similar to that of the healthy adults and more dissimilar to adults with active neuroinflammatory disorders, analyzed in the same lab (Additional file 1: Figure S1). A second limitation of this study, which we protected against by correcting for the false detection rate, was the large number of comparisons which may lead to spurious results. A third limitation was the detection limits of the assays, and high rates of out-of-range values in several cases required us to model the proportion out-of-range rather than the mean value. It is worth noting that a difference in rate of out-of-range values is similar to, but not the same as, a difference in mean values. This type of dichotomization does reduce the power of a statistical test to detect relationships, and assays with different sensitivities may have produced different results. Finally, although the majority of participants contributed at least two serum samples to the longitudinal analysis, it is possible that the degree of data missingness may have influenced the results.

## Conclusions

In conclusion, this is the first longitudinal study of the profile of serum immune mediators in children with ASD compared to controls. The large sample size and repeated measures design of this study are significant strengths, as the power to detect differences between groups is increased when error variance due to individual differences is reduced. Further, we explicitly modeled the potential confounding effects of age in this sample, which spanned from 2–8 years, and reduced the risk spurious results due to age-related differences in immune profiles. We also utilized groups matched on sex, and controlled for sex in our statistical model, given some data suggesting that males and females respond differently to in utero inflammation [77]. Finally, this is the only longitudinal study of chemokines and cytokines in the CSF of children with ASD. These data provide important information about the lack of relationship between central and peripheral immune markers, signaling that caution should be taken when interpreting the available studies implicating current immune dysfunction in the phenomenology of ASD, as few have included direct measures of CNS status.

## Additional file

Additional file 1: Table S1. Basic laboratory features. **Table S2.** Serum descriptive data by age bands and diagnostic group. **Table S3.** CSF descriptive data by age cohort. **Table S4.** Correspondence between serum and CSF values in AUT sample (*n* = 54). **Figure S1.** Profile of selected CSF immune mediators in AUT and two adult comparison groups, median values and interquartile range. (DOCX 209 kb)

## Abbreviations

ASD: Autism spectrum disorder
AUT: Autism study group
CNS: Central nervous system
CSF: Cerebrospinal fluid
LP: Lumbar puncture
TYP: Typically developing study group
