# Supplementary material for: Serum and cerebrospinal fluid immune mediators in children with autistic disorder: a longitudinal study
Source: Mol Autism. 2017 Jan 5;8:1. doi: 10.1186/s13229-016-0115-7 (PMC5217649; doi:10.1186/s13229-016-0115-7)
Supplement: Additional file 1: Table S1. — Basic laboratory features. Table S2. Serum descriptive data by age bands and diagnostic group. Table S3. CSF descriptive data by age cohort. Table S4. Correspondence between serum and CSF values in AUT sample (n = 54). Figure S1. Profile of selected CSF immune mediators in AUT and two adult comparison groups, median values and interquartile range. (DOCX 209 kb) [file 13229_2016_115_MOESM1_ESM.docx]

Additional file 1

**Table S1**. Basic laboratory features

|  | **Full Sample** | | | | | | |  | **Subsample with CSF** | | |
| --- | --- | --- | --- | --- | --- | --- | --- | --- | --- | --- | --- |
|  | **AUT, *N*=104** | | |  | **TYP, *N*=54** | | |  | **AUT, *N*=67** | | |
|  | *n* | IQR | *M* ± *SD* |  | *n* | IQR | *M* ± *SD* |  | *n* | IQR | *M* ± *SD* |
| Serum |  |  |  |  |  |  |  |  |  |  |  |
| WBC Count | 101 | (6.32 – 9.57) | 8.15 ± 2.31 |  | 52 | (6.08 – 8.325) | 7.16 ± 1.73 |  | 65 | (6.76 – 9.71) | 8.42 ± 2.37 |
| Platelet Count | 102 | (292 – 381) | 340.73 ± 75.73 |  | 18 | (272 – 350) | 311.17 ± 58.90 |  | 65 | (284 – 375) | 330.80 ± 73.74 |
| Polys (%) | 71 | (31.5 – 46.5) | 39.37 ± 10.09 |  | 18 | (26.7 – 38) | 35.42 ± 10.17 |  | 44 | (29.3 – 45) | 37.69 ± 10.76 |
| Lymphocytes (%) | 98 | (40 – 54) | 47.35 ± 10.6 |  | 52 | (42 – 55.5) | 48.26 ± 11.18 |  | 63 | (41 – 56) | 47.88 ± 10.48 |
| Monocytes (%) | 98 | (7.8 – 10) | 8.87 ± 2.7 |  | 52 | (7 – 9) | 8.07 ± 2.13 |  | 63 | (8 – 10) | 9.10 ± 2.63 |
| Eosinophils (%) | 98 | (2 – 5) | 3.98 ± 3.38 |  | 52 | (2 – 3.95) | 3.05 ± 2.37 |  | 63 | (2 – 4) | 3.73 ± 3.40 |
| IgG, mg/dL | 95 | (646 – 1010) | 838.07 ± 251.71 |  | 52 | (601 – 831.5) | 760.94 ± 188.37 |  | 62 | (616 – 929) | 774.52 ± 252.22 |
| IgM, mg/dL | 95 | (60 – 100) | 83.95 ± 35.52 |  | 53 | (60 – 104) | 84.89 ± 33.78 |  | 62 | (58 – 100) | 80.73 ± 37.48 |
| IgA, mg/dL | 95 | (53 – 100) | 88.05 ± 51.82 |  | 52 | (53 – 113) | 86.81 ± 46.77 |  | 62 | (50 – 91) | 78.48 ± 46.26 |
| CSF |  |  |  |  |  |  |  |  |  |  |  |
| WBC Count |  |  |  |  |  |  |  |  | 63 | (0 – 1) | 0.79 ± 1.03 |
| Albumin Quotient |  |  |  |  |  |  |  |  | 61 | (1.96 – 2.77) | 2.62 ± 1.60 |
| IgG, mg/dL |  |  |  |  |  |  |  |  | 60 | (<LOD – 0.95) | –– |
| IgG Index |  |  |  |  |  |  |  |  | 60 | (<LOD – 0.55) | –– |

Note: <LOD=less than level of detection. LOD was 0.90 for CSF IgG. No significant differences were observed between AUT and TYP on laboratory features. Using age-based norms, values were flagged as low or high. The rate of flags did not differ between groups.

**Table S2A**. Serum Descriptive Data by Age Bands and Diagnostic Group,

|  | **Autism** | | | | | | | | |
| --- | --- | --- | --- | --- | --- | --- | --- | --- | --- |
|  | **Age 2-3.99 Years (*n*=43)** | | | **Age 4-5.99 Years (*n*=75)** | | | **Age 6-7.99 Years (*n*=47)** | | |
|  | **% Out of Range** | **Median** | **IQR** | **% Out of Range** | **Median** | **IQR** | **% Out of Range** | **Median** | **IQR** |
| IL-1α | 23% | 14.69 | (7.9-52.32) | 16% | 17.89 | (6.08-62.07) | 6% | 19.55 | (12.01-37.97) |
| IL-1RA | 56% | <LOD | (<LOD-105.28) | 51% | <LOD | (<LOD-144.56) | 49% | 32.95 | (<LOD-121.63) |
| IL-1β | 60% | <LOD | (<LOD-9.98) | 53% | <LOD | (<LOD-15.55) | 51% | <LOD | (<LOD-13.68) |
| IL-2 | 40% | 3.19 | (<LOD-5.91) | 31% | 3.08 | (<LOD-6.64) | 30% | 3.52 | (<LOD-7.6) |
| sIL-2RA | 19% | 42.76 | (21.45-74.9) | 27% | 35.39 | (<LOD-80.42) | 51% | <LOD | (<LOD-55.32) |
| IL-3 | 40% | 0.87 | (<LOD-1.47) | 39% | 0.86 | (<LOD-1.23) | 43% | 0.76 | (<LOD-1.54) |
| IL-4 | 37% | 7.58 | (<LOD-18.01) | 23% | 8.91 | (3.38-27.55) | 26% | 12.52 | (<LOD-24.3) |
| IL-5 | 7% | 1.31 | (0.41-3.08) | 12% | 1.19 | (0.58-3.19) | 6% | 1.16 | (0.61-2.75) |
| IL-6 | 42% | 4.39 | (<LOD-8.83) | 35% | 5.10 | (<LOD-11.83) | 30% | 5.19 | (<LOD-8.49) |
| IL-7 | 19% | 4.80 | (2.43-8.31) | 9% | 6.49 | (4.2-9.5) | 9% | 6.99 | (4.02-10.05) |
| IL-9 | 5% | 1.80 | (0.87-5.77) | 4% | 2.05 | (1.07-4.88) | 4% | 2.64 | (1.21-3.95) |
| IL-10 | 23% | 7.68 | (2.3-11.76) | 31% | 6.95 | (<LOD-16.76) | 32% | 5.52 | (<LOD-12.09) |
| IL-12p40 | 19% | 29.99 | (18.24-76.18) | 19% | 28.75 | (17.45-67.75) | 19% | 32.56 | (24.23-70.95) |
| IL-12p70 | 16% | 3.94 | (1.65-7) | 11% | 5.20 | (2.66-9.24) | 17% | 6.60 | (3.6-14.21) |
| IL-13 | 44% | 11.84 | (<LOD-47.92) | 43% | 7.31 | (<LOD-45.39) | 36% | 9.58 | (<LOD-23.89) |
| IL-15 | 60% | <LOD | (<LOD-8.61) | 59% | <LOD | (<LOD-13.06) | 51% | <LOD | (<LOD-11.88) |
| IL-17 | 9% | 3.98 | (1.74-8.78) | 5% | 5.30 | (2.2-8.97) | 6% | 6.32 | (2.87-13.24) |
| IFNα2 | 2% | 41.58 | (26.84-79.72) | 7% | 41.08 | (27.4-85.45) | 2% | 48.70 | (26.68-76.58) |
| IFNγ | 2% | 9.35 | (6.55-25.13) | 3% | 14.54 | (7.02-28.93) | 0% | 20.76 | (5.97-30.69) |
| TNF α | 0% | 14.62 | (11.78-19.61) | 0% | 13.80 | (10.27-19.56) | 0% | 12.20 | (9.33-15.84) |
| TNF β | 40% | 5.78 | (<LOD-14.74) | 25% | 3.43 | (<LOD-16.74) | 28% | 4.89 | (<LOD-10.65) |
| TGF α | 5% | 5.18 | (3.58-7.86) | 5% | 5.29 | (2.54-8.81) | 6% | 4.45 | (3.16-6.41) |
| EGF | 0% | 43.35 | (25.47-89.86) | 4% | 44.75 | (24.79-72.63) | 4% | 38.31 | (28.36-66.59) |
| G-CSF | 0% | 42.75 | (26.18-74.33) | 1% | 42.28 | (27.96-62.72) | 0% | 34.62 | (26.41-62.52) |
| GM-CSF | 0% | 75.31 | (35.67-158.48) | 3% | 65.17 | (36.41-127.19) | 0% | 59.37 | (35.3-253.66) |
| VEGF | 0% | 185.56 | (99.7-288.55) | 3% | 229.25 | (157.36-334.84) | 0% | 206.75 | (128.92-346.23) |
| FGF-2 | 7% | 50.71 | (34.28-65.13) | 5% | 58.77 | (39.2-76.1) | 6% | 56.44 | (43.01-73.73) |
| FLT-3L | 77% | <LOD | (<LOD-<LOD) | 55% | <LOD | (<LOD-27.18) | 49% | 17.09 | (<LOD-24.62) |
| sCD40L | 67% | >LOD | (2923.63->LOD) | 72% | >LOD | (1496.02->LOD) | 68% | >LOD | (1696.54->LOD) |
| CCL2 (MCP-1) | 0% | 423.25 | (305.82-559.92) | 0% | 483.32 | (302.52-612.7) | 0% | 423.63 | (263.93-486.96) |
| CCL3 (MIP-1 α) | 7% | 6.93 | (4.39-13.18) | 7% | 7.51 | (4.82-14.67) | 4% | 7.23 | (5.77-12.03) |
| CCL4 (MIP-1 β) | 0% | 57.81 | (37.5-80.4) | 1% | 59.12 | (37.41-80.16) | 0% | 49.36 | (34.82-70.73) |
| CCL7 (MCP-3) | 26% | 16.48 | (<LOD-33.88) | 17% | 15.03 | (9.8-35.17) | 11% | 15.25 | (8.69-20.88) |
| CCL11 (EOTAXIN) | 2% | 132.86 | (101.7-151.3) | 1% | 128.66 | (93.12-161.46) | 0% | 124.05 | (77.87-178.18) |
| CCL22 (MDC) | 0% | 1898.92 | (1459.13-2142.64) | 0% | 1631.51 | (1328.47-1951.93) | 0% | 1500.48 | (1199.82-1836.35) |
| CXCL1 (GRO) | 0% | 618.92 | (475.79-976.71) | 0% | 723.11 | (506.82-896.1) | 0% | 619.42 | (444.12-913.93) |
| CXCL8 (IL-8) | 0% | 10.19 | (6.97-17.62) | 0% | 12.63 | (8.53-20.08) | 0% | 12.18 | (8.8-17.04) |
| CXCL10 (IP-10) | 0% | 261.23 | (197.45-511.32) | 0% | 289.93 | (219.57-460.07) | 0% | 259.59 | (205.31-383.89) |
| CX3CL1 (FRACTALKINE) | 33% | 54.55 | (<LOD-120.21) | 23% | 59.63 | (40.07-130.83) | 28% | 80.24 | (<LOD-116.95) |

Note: IQR=interquartile range (25^th^-75^th^ percentile). LOD=level of detection. Refer to Table 3 for the detection limit of each analyte. Units are pg/mL. These data do not directly correspond to the statistical tests presented in the main text, in which the time metric was chronological age (not visit or age-band). These tables are a simple summary of the data. The design of this study was accelerated longitudinal, meaning that the data were not analyzed by visit, as each visit did not correspond to a particular age range. However, the observations had to be collated into age-based categories for presentation. Thus, each observation from a subject was classified into an age-band (2-3.99 years, 4-5.99 years, and 6-7.99 years). If a subject had more than one visit during an age band, only the first visit was used, so a subject is not represented more than once per age band (but may appear in more than one age band).

**Table S2B**. Serum Descriptive Data by Age Bandst and Diagnostic Group,

|  | **Typical** | | | | | | | | |
| --- | --- | --- | --- | --- | --- | --- | --- | --- | --- |
|  | **Age 2-3.99 Years (*n*=34)** | | | **Age 4-5.99 Years (*n*=34)** | | | **Age 6-7.99 Years (*n*=18)** | | |
|  | **% Out of Range** | **Median** | **IQR** | **% Out of Range** | **Median** | **IQR** | **% Out of Range** | **Median** | **IQR** |
| IL-1α | 18% | 19.89 | (11.33-45.54) | 15% | 15.83 | (12.03-37.14) | 11% | 24.30 | (13.365-31.32) |
| IL-1RA | 44% | 80.32 | (<LOD-146.87) | 44% | 30.19 | (<LOD-260) | 56% | <LOD | (<LOD-115.66) |
| IL-1β | 53% | <LOD | (<LOD-16.31) | 38% | 4.80 | (<LOD-25.06) | 50% | <LOD | (<LOD-15.15) |
| IL-2 | 24% | 2.69 | (1.14-7.28) | 21% | 3.28 | (1.88-8.89) | 22% | 3.07 | (1.84-6.8) |
| sIL-2RA | 29% | 35.48 | (<LOD-66.42) | 29% | 28.83 | (<LOD-51.8) | 67% | <LOD | (<LOD-52.65) |
| IL-3 | 62% | <LOD | (<LOD-1.86) | 47% | 1.24 | (<LOD-1.73) | 61% | <LOD | (<LOD-1.17) |
| IL-4 | 29% | 6.40 | (<LOD-21.39) | 21% | 8.28 | (5.2-14.35) | 44% | 6.23 | (<LOD-12.09) |
| IL-5 | 9% | 0.72 | (0.33-1.96) | 12% | 1.08 | (0.67-1.77) | 17% | 1.28 | (0.77-2.95) |
| IL-6 | 35% | 4.50 | (<LOD-9.22) | 26% | 4.06 | (<LOD-7.93) | 39% | 3.28 | (<LOD-16.57) |
| IL-7 | 15% | 7.17 | (4.44-10.69) | 9% | 8.30 | (5.15-11.51) | 17% | 6.11 | (2.69-7.99) |
| IL-9 | 6% | 1.93 | (1.29-4.38) | 9% | 2.26 | (1.26-5.86) | 17% | 2.45 | (1.55-7.35) |
| IL-10 | 26% | 4.20 | (<LOD-12.22) | 26% | 6.18 | (<LOD-23.63) | 22% | 2.20 | (1.43-9.71) |
| IL-12p40 | 12% | 41.15 | (21.95-80.67) | 6% | 37.37 | (26.16-65.84) | 11% | 34.08 | (6.49-61.63) |
| IL-12p70 | 12% | 4.84 | (2.76-8.79) | 12% | 7.61 | (5.19-14.49) | 28% | 5.21 | (<LOD-7.86) |
| IL-13 | 32% | 12.27 | (<LOD-33.73) | 26% | 11.49 | (<LOD-34.71) | 44% | 12.54 | (<LOD-28.98) |
| IL-15 | 50% | <LOD | (<LOD-9.44) | 50% | <LOD | (<LOD-16.35) | 50% | <LOD | (<LOD-5) |
| IL-17 | 6% | 2.66 | (0.94-10.5) | 3% | 6.18 | (3.74-15.92) | 6% | 7.51 | (2.63-13.21) |
| IFNα2 | 3% | 37.84 | (24.61-72.88) | 0% | 46.81 | (29.06-81.71) | 6% | 54.83 | (24.4-61.28) |
| IFNγ | 6% | 11.67 | (5.31-28.26) | 0% | 14.46 | (9.92-51.16) | 6% | 18.24 | (7.14-36.43) |
| TNF α | 0% | 15.83 | (12.41-18.84) | 0% | 14.91 | (10.73-16.9) | 0% | 11.21 | (7.07-13.54) |
| TNF β | 21% | 3.92 | (1.76-11.13) | 18% | 7.29 | (3.5-18.65) | 28% | 4.73 | (<LOD-12.25) |
| TGF α | 18% | 5.13 | (2.9-7.8) | 12% | 4.39 | (2.3-6.51) | 39% | 3.61 | (<LOD-5.65) |
| EGF | 9% | 31.41 | (15.9-52.98) | 3% | 28.41 | (20.13-43.62) | 11% | 31.69 | (25.335-40.08) |
| G-CSF | 0% | 47.87 | (32.49-86.05) | 3% | 51.95 | (35.01-70.28) | 0% | 36.34 | (19.53-60.42) |
| GM-CSF | 6% | 49.90 | (33.97-98.54) | 0% | 95.85 | (52.33-168.05) | 0% | 113.53 | (91.41-203.31) |
| VEGF | 3% | 151.51 | (119.7-321.73) | 3% | 159.40 | (96.47-215.93) | 6% | 172.73 | (87.33-292.54) |
| FGF-2 | 9% | 53.55 | (41.48-69.31) | 3% | 52.44 | (40.99-74.22) | 17% | 42.00 | (24.3-68.51) |
| FLT-3L | 50% | <LOD | (<LOD-12.44) | 44% | 13.20 | (<LOD-18.06) | 67% | <LOD | (<LOD-17.1) |
| sCD40L | 38% | 1916.83 | (652.13-5540.38) | 38% | 2681.33 | (1472.76-5282.85) | 28% | 866.05 | (619.36-2535.79) |
| CCL2 (MCP-1) | 0% | 358.67 | (228.01-608.78) | 0% | 403.66 | (292.34-557.05) | 0% | 258.98 | (161.05-473.28) |
| CCL3 (MIP-1 α) | 9% | 7.25 | (5.28-13.26) | 3% | 7.40 | (4.97-12.07) | 11% | 8.51 | (4.21-17.11) |
| CCL4 (MIP-1 β) | 0% | 53.58 | (40.37-76.81) | 0% | 57.01 | (39.42-71.9) | 0% | 45.98 | (29.83-70.32) |
| CCL7 (MCP-3) | 15% | 16.46 | (11.11-23.79) | 21% | 17.89 | (13.27-31.96) | 22% | 14.18 | (8.82-21.04) |
| CCL11 (EOTAXIN) | 3% | 120.00 | (84.8-165.9) | 0% | 135.54 | (98.44-191.74) | 0% | 110.24 | (90.17-140.41) |
| CCL22 (MDC) | 3% | 1587.04 | (1156.67-1953) | 0% | 1540.53 | (1258.93-1914.15) | 0% | 1365.47 | (967.92-1772.27) |
| CXCL1 (GRO) | 0% | 577.76 | (493.74-886.23) | 0% | 618.53 | (534.25-766.94) | 0% | 476.64 | (387.29-747.97) |
| CXCL8 (IL-8) | 0% | 8.89 | (6.91-17.28) | 0% | 13.21 | (8.35-21.35) | 0% | 12.26 | (3.45-19.11) |
| CXCL10 (IP-10) | 0% | 239.87 | (181.79-340.12) | 0% | 255.91 | (207-358.73) | 0% | 269.75 | (189.84-377.1) |
| CX3CL1 (FRACTALKINE) | 15% | 58.75 | (39.93-92.26) | 18% | 77.80 | (53.63-104.32) | 33% | 89.44 | (<LOD-108.92) |

Note: IQR=interquartile range (25^th^-75^th^ percentile). LOD=level of detection. Refer to Table 3 for the detection limit of each analyte. Units are pg/mL. These data do not directly correspond to the statistical tests presented in the main text, in which the time metric was chronological age (not visit or age-band). These tables are a simple summary of the data. The design of this study was accelerated longitudinal, meaning that the data were not analyzed by visit, as each visit did not correspond to a particular age range. However, the observations had to be collated into time-based categories for presentation. Thus, each observation from a subject was classified into an age-band (2-3.99 years, 4-5.99 years, and 6-7.99 years). If a subject had more than one visit during an age band, only the first visit was used, so a subject is not represented more than once per age band (but may appear in more than one age band).

**Table S3**. CSF Descriptive Data by Age Cohort

|  | **Age 2-4 Years (N=47)** | | | **Age 4-6 Years (N=33)** | | | **Age 6-8 Years (N=15)** | | |
| --- | --- | --- | --- | --- | --- | --- | --- | --- | --- |
|  | **% Out of Range** | **Median** | **IQR** | **% Out of Range** | **Median** | **IQR** | **% Out of Range** | **Median** | **IQR** |
| IL-1α | 4% | 2.03 | (1.46-3.94) | 15% | 2.7 | (1.505-3.875) | 0% | 1.96 | (1.15-3.7) |
| IL-1RA | 74% | <LOD | (<LOD-2.875) | 82% | <LOD | (<LOD-<LOD) | 73% | <LOD | (<LOD-2.705) |
| IL-1β | 77% | <LOD | (<LOD-<LOD) | 73% | <LOD | (<LOD-0.23) | 80% | <LOD | (<LOD-<LOD) |
| IL-2 | 74% | <LOD | (<LOD-0.29) | 91% | <LOD | (<LOD-<LOD) | 60% | <LOD | (<LOD-0.18) |
| sIL-2RA | 53% | <LOD | (<LOD-1.49) | 70% | <LOD | (<LOD-0.93) | 47% | 0.65 | (<LOD-1.025) |
| IL-3 | 49% | 0.72 | (<LOD-0.8) | 64% | <LOD | (<LOD-0.715) | 33% | 0.675 | (<LOD-0.8) |
| IL-4 | 94% | <LOD | (<LOD-<LOD) | 91% | <LOD | (<LOD-<LOD) | 100% | <LOD | (<LOD-<LOD) |
| IL-5 | 9% | 0.57 | (0.23-0.67) | 6% | 0.56 | (0.35-0.66) | 7% | 0.635 | (0.15-0.71) |
| IL-6 | 94% | <LOD | (<LOD-<LOD) | 91% | <LOD | (<LOD-<LOD) | 87% | <LOD | (<LOD-<LOD) |
| IL-7 | 64% | <LOD | (<LOD-1.96) | 79% | <LOD | (<LOD-<LOD) | 73% | <LOD | (<LOD-2.33) |
| IL-9 | 0% | 1.24 | (1.08-1.44) | 0% | 1.45 | (1.23-1.61) | 0% | 1.68 | (1.23-1.9) |
| IL-10 | 34% | 1.03 | (<LOD-1.5) | 27% | 0.89 | (<LOD-1.24) | 20% | 1.09 | (0.895-1.445) |
| IL-12p40 | 51% | <LOD | (<LOD-4.27) | 67% | <LOD | (<LOD-3.15) | 33% | 2.45 | (<LOD-4.27) |
| IL-12p70 | 85% | <LOD | (<LOD-<LOD) | 91% | <LOD | (<LOD-<LOD) | 93% | <LOD | (<LOD-<LOD) |
| IL-13 | 96% | <LOD | (<LOD-<LOD) | 97% | <LOD | (<LOD-<LOD) | 100% | <LOD | (<LOD-<LOD) |
| IL-15 | 0% | 1.48 | (0.77-2.02) | 0% | 1.39 | (0.98-1.84) | 0% | 1.62 | (1.43-2.41) |
| IL-17 | 91% | <LOD | (<LOD-<LOD) | 91% | <LOD | (<LOD-<LOD) | 93% | <LOD | (<LOD-<LOD) |
| IFNα2 | 6% | 5.515 | (3.61-6.875) | 0% | 4.47 | (3.02-5.8) | 0% | 5.18 | (3.99-6.69) |
| IFNγ | 53% | <LOD | (<LOD-0.47) | 70% | <LOD | (<LOD-0.49) | 40% | 0.21 | (<LOD-0.35) |
| TNF α | 0% | 0.74 | (0.61-0.94) | 0% | 0.66 | (0.57-0.83) | 0% | 0.7 | (0.52-0.92) |
| TNF β | 49% | 0.11 | (<LOD-0.22) | 61% | <LOD | (<LOD-0.16) | 53% | <LOD | (<LOD-0.37) |
| TGF α | 0% | 3.08 | (2.34-4) | 0% | 2.93 | (2.34-3.76) | 0% | 3.33 | (2.45-4.02) |
| EGF | 100% | <LOD | (<LOD-<LOD) | 100% | <LOD | (<LOD-<LOD) | 100% | <LOD | (<LOD-<LOD) |
| G-CSF | 0% | 4.91 | (3.67-6.77) | 3% | 3.665 | (2.24-5.795) | 0% | 4.22 | (3.12-5.49) |
| GM-CSF | 0% | 2.45 | (1.98-3.05) | 0% | 2.36 | (1.7-2.94) | 0% | 2.6 | (2.31-3.19) |
| VEGF | 87% | <LOD | (<LOD-<LOD) | 88% | <LOD | (<LOD-<LOD) | 93% | <LOD | (<LOD-<LOD) |
| FGF-2 | 66% | <LOD | (<LOD-11.19) | 73% | <LOD | (<LOD-7.48) | 60% | <LOD | (<LOD-6.51) |
| FLT-3L | 0% | 11.02 | (8.8-14.29) | 0% | 12.08 | (10.28-14.39) | 0% | 14.24 | (12.51-16.63) |
| sCD40L | 11% | 17.83 | (13.4-25.99) | 3% | 19.705 | (13.54-29.445) | 7% | 14.76 | (9.53-31.74) |
| CCL2  (MCP-1) | 0% | 523.9 | (468.72-568.64) | 0% | 528.44 | (449.77-628.49) | 0% | 522.88 | (484.39-575.79) |
| CCL3  (MIP-1 α) | 47% | 2.07 | (<LOD-2.85) | 39% | 1.43 | (<LOD-2.145) | 60% | <LOD | (<LOD-2.98) |
| CCL4  (MIP-1 β) | 45% | 3.44 | (<LOD-5.38) | 52% | <LOD | (<LOD-5.48) | 53% | <LOD | (<LOD-8.37) |
| CCL7  (MCP-3) | 57% | <LOD | (<LOD-6.195) | 61% | <LOD | (<LOD-5.72) | 47% | 3.61 | (<LOD-4.57) |
| CCL11 (EOTAXIN) | 94% | <LOD | (<LOD-<LOD) | 94% | <LOD | (<LOD-<LOD) | 80% | <LOD | (<LOD-<LOD) |
| CCL22  (MDC) | 13% | 13.08 | (8.8-15.99) | 24% | 10.84 | (6.81-14.77) | 13% | 8.8 | (6.81-13.08) |
| CXCL1  (GRO) | 2% | 10.235 | (8.14-13.15) | 3% | 9.955 | (7.68-12.64) | 0% | 10.89 | (8.22-13.96) |
| CXCL8  (IL-8) | 0% | 15 | (12.65-20.41) | 0% | 15.73 | (14.38-18.28) | 0% | 17.26 | (14.3-20.13) |
| CXCL10  (IP-10) | 0% | 301.21 | (224.11-494.8) | 0% | 311.7 | (213.92-509.18) | 0% | 258.8 | (194.8-317.16) |
| CX3CL1 (FRACTALKINE) | 0% | 72.41 | (63.62-93.48) | 0% | 87.38 | (67.41-98.46) | 0% | 85.97 | (72.43-91.43) |

Note: IQR=interquartile range (25^th^-75^th^ percentile). LOD=level of detection. Units are pg/mL. Table reflects all available data from each participant such that the total N of the table sums to the number of observations rather than the number of subjects. Only the first visit was used when a participant had more than one observation per age cohort.

**Table S4**. Correspondence between serum and CSF values in AUT sample (n=54)

|  | **Outside Range of Detection** | | | |  | **Spearman Correlation** | | |
| --- | --- | --- | --- | --- | --- | --- | --- | --- |
|  | **CSF Only** | **Serum Only** | **Both** | **Neither** |  | **n** | **r (95% CI)** | **FDR-Adjusted p** |
| **Cytokines** |  |  |  |  |  |  |  |  |
| IL-1A | 7% | 20% | 2% | 70% |  | 42 | .24 (-.09 – .52) | .29 |
| IL-IRA | 35% | 9% | 43% | 13% |  |  |  |  |
| IL-1B | 26% | 11% | 46% | 17% |  |  |  |  |
| IL-2 | 52% | 4% | 31% | 13% |  |  |  |  |
| sIL-2RA | 46% | 7% | 17% | 30% |  |  |  |  |
| IL-3 | 37% | 15% | 22% | 26% |  |  |  |  |
| IL-4 | 61% | 0% | 30% | 9% |  |  |  |  |
| IL-5 | 11% | 7% | 0% | 81% |  | 48 | .04 (-.26 – .33) | .81 |
| IL-6 | 54% | 0% | 37% | 9% |  |  |  |  |
| IL-7 | 57% | 6% | 11% | 26% |  |  |  |  |
| IL-9 | 0% | 4% | 0% | 96% |  | 52 | .07 (-.20 – .34) | .69 |
| IL-10 | 26% | 22% | 6% | 46% |  | 37 | .37 (-.04 – .66) | .21 |
| IL-12p40 | 56% | 7% | 9% | 28% |  |  |  |  |
| IL-12p70 | 81% | 0% | 7% | 11% |  |  |  |  |
| IL-13 | 57% | 0% | 39% | 4% |  |  |  |  |
| IL-15 | 0% | 59% | 0% | 41% |  |  |  |  |
| IL-17 | 85% | 0% | 7% | 7% |  |  |  |  |
| IFNα2 | 4% | 2% | 0% | 94% |  | 52 | .08 (-.20 – .35) | .69 |
| IFNγ | 57% | 0% | 4% | 39% |  |  |  |  |
| TNFα | 0% | 0% | 0% | 100% |  | 54 | .32 (.05 – .54) | .07 |
| TNFβ | 35% | 9% | 26% | 30% |  |  |  |  |
| TGFα | 0% | 4% | 0% | 96% |  | 52 | -.15 (-.41 – .13) | .43 |
| **Growth Factors** |  |  |  |  |  |  |  |  |
| EGF | 100% | 0% | 0% | 0% |  |  |  |  |
| G-CSF | 2% | 0% | 0% | 98% |  | 53 | .21 (-.07 – .45) | .29 |
| GM-CSF | 0% | 2% | 0% | 98% |  | 53 | -.07 (-.34 – .20) | .69 |
| VEGF | 94% | 0% | 0% | 6% |  |  |  |  |
| FGF-2 | 69% | 0% | 6% | 26% |  |  |  |  |
| FLT-3L | 0% | 70% | 0% | 30% |  |  |  |  |
| sCD40L | 2% | 69% | 7% | 22% |  |  |  |  |
| **Chemokines** |  |  |  |  |  |  |  |  |
| CCL2 (MCP-1) | 0% | 0% | 0% | 100% |  | 54 | .54 (.31 – .70) | .003 |
| CCL3 (MIP-1α) | 43% | 2% | 7% | 48% |  |  |  |  |
| CCL4 (MIP-1β) | 52% | 0% | 0% | 48% |  |  |  |  |
| CCL7 (MCP-3) | 46% | 4% | 15% | 35% |  |  |  |  |
| CCL11 (EOTAXIN) | 93% | 0% | 2% | 6% |  |  |  |  |
| CCL22 (MDC) | 17% | 0% | 0% | 83% |  | 45 | .45 (.17 – .65) | .01 |
| CXCL1 (GRO) | 2% | 0% | 0% | 98% |  | 53 | .20 (-.08 – .44) | .29 |
| CXCL8 (IL-8) | 0% | 0% | 0% | 100% |  | 54 | .06 (-.21 – .33) | .69 |
| CXCL10 (IP-10) | 0% | 0% | 0% | 100% |  | 54 | .57 (.35 – .72) | .003 |
| CX3CL1 (FRACTALKINE) | 0% | 26% | 0% | 74% |  | 40 | .18 (-.14 – .47) | .43 |

Note: Rates outside the range of detection sum to 100%; total rate for CSF is CSF Only plus Both, and total rate for serum is Serum Only plus Both. Spearman correlations were calculated with listwise deletion when either the CSF or serum value was out of range; correlations were not calculated where the resulting *n* was less than 70% of the sample (*n*=38).

**Figure S1**. Profile of selected CSF Immune Mediators in AUT and two adult comparison groups, median values and interquartile range


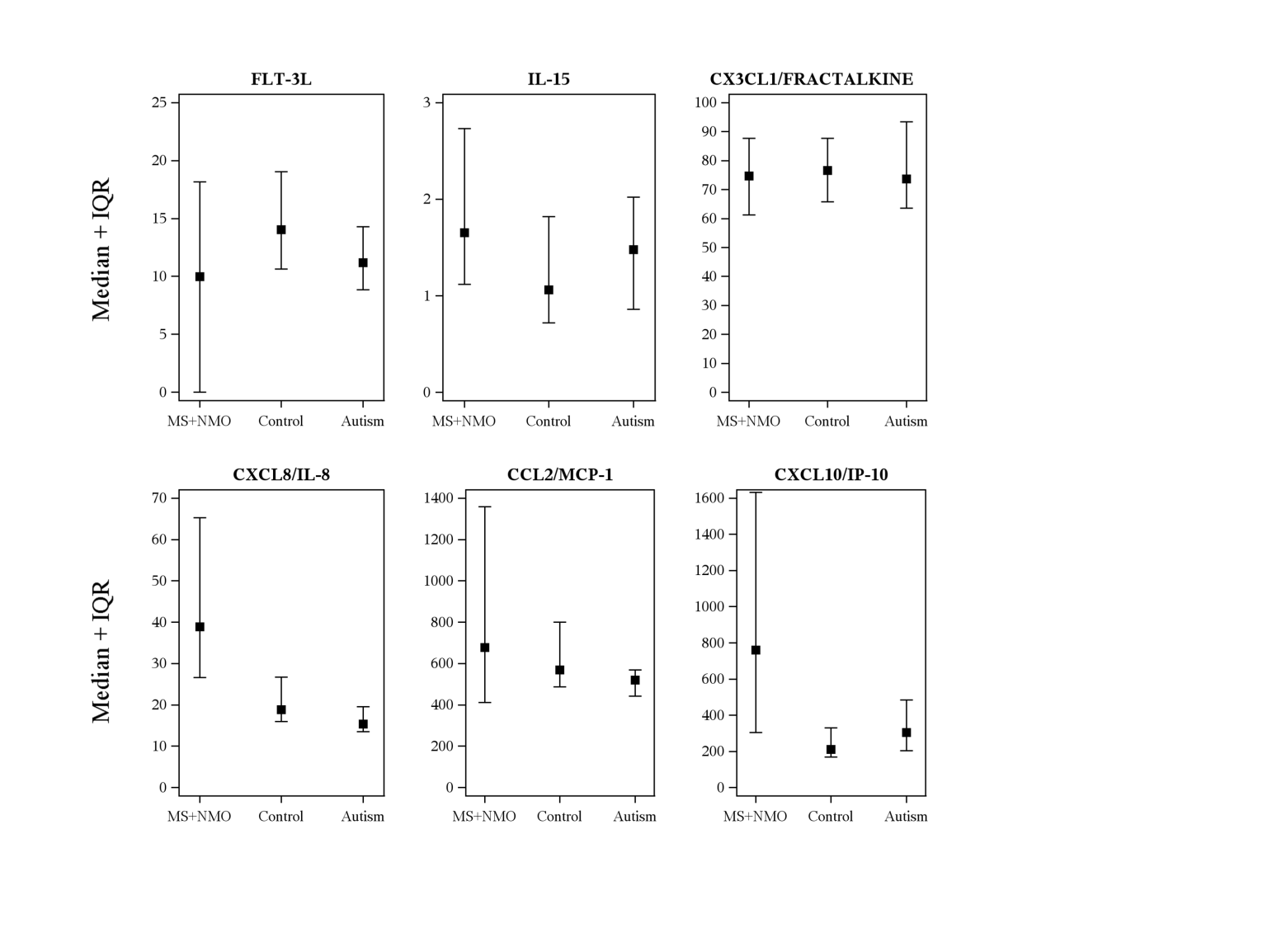


Note: Y axis is in pg/mL. IQR=interquartile range. MS+NMO=combined sample of adults with multiple sclerosis (*n*=12) and neormyelitis optica (*n*=11). Controls=healthy adults (*n*=13). Autism=AUT group from present study (*n*=67).
